# Comparable diagnostic accuracy of SARS-CoV-2 Spike RBD and N-specific IgG tests to determine pre-vaccination nation-wide baseline seroprevalence in Mexico

**DOI:** 10.1038/s41598-022-22146-8

**Published:** 2022-10-26

**Authors:** Jesús Martínez-Barnetche, Martha Carnalla, Carlos Gaspar-Castillo, Ana Basto-Abreu, Ricardo Lizardi, Rodrigo Aparicio Antonio, Irma López Martinez, Anais Cortes Escamilla, Octavio T. Ramirez, Laura A. Palomares, Daniel Barreto-Cabrera, Juan Carlos Rivera-Castro, Carlos Segura-Sánchez, Mauricio Hernández Ávila, Tonatiuh Barrientos-Gutiérrez, Celia M. Alpuche Aranda

**Affiliations:** 1 Centro de Investigación Sobre Enfermedades Infecciosas, Instituto Nacional de Salud Pública, Cuernavaca, Morelos, Mexico; 2 Centro de Investigación en Salud Poblacional, Instituto Nacional de Salud Pública, Cuernavaca, Mexico; 3 Instituto de Diagnóstico y Referencia Epidemiológicos, Secretaria de Salud, Mexico City, Mexico; 4 Instituto de Biotecnología, Universidad Nacional Autónoma de México, Cuernavaca, Morelos, Mexico; 5 Dirección General de Prestaciones Médicas, Instituto Mexicano del Seguro Social, Mexico City, Mexico

**Keywords:** Epidemiology, Population screening

## Abstract

A major challenge for developing countries during the COVID-19 pandemic is affordable and adequate monitoring of disease progression and population exposure as the primary source relevant epidemiological indicators. Serology testing enables assessing population exposure and to guide vaccination strategies but requires rigorous accuracy validation before population-wide implementation. We adapted a two-step ELISA protocol as a single-step protocol for detection of IgG against the Receptor Binding Domain (RBD) of SARS-CoV-2 spike protein and compared its diagnostic accuracy with a commercial immunoassay anti-nucleoprotein IgG. Both methods yielded adequate and comparable diagnostic accuracy after 3 weeks post-symptom onset and were implemented in a nation-wide population based serological survey during August–November 2020. Anti-RBD National seroprevalence was 23.6%, 1.3% lower, but not significantly, than for anti-N. Double positive seroprevalence was 19.7%. Anti-N single-positive seroprevalence was 3.72% and anti-RBD single-positive seroprevalence was 1.98%. Discrepancies in the positivity to either single marker may be due to different kinetics of each antibody marker as well as the heterogeneity of the sampling time in regards to local epidemic waves. Baseline single positivity prevalence will be useful to assess the serological impact of vaccination and natural infection in further serosurveillance efforts.

## Introduction

Developing countries have faced important challenges to respond to the COVID-19 pandemic. Access to low-cost options to assess the extent and occurrence of infection has been a key limitation for epidemic monitoring. Serology testing is used to measure previous population exposure, an approximation to population immunity and provides adequate denominators for the estimation of attack and lethality rates^1^.

Validating serologic tests is key to adequately inform seroprevalence. Serologic cross-reactivity between endemic HuCoV’s and SARS-CoV is well documented^2–4^; thus, the risk of false-positives as a result of previous HuCoV exposure has to be considered to validate serologic assays during the current COVID-19 pandemic^5^. Beyond cross-reactivity, other factors such as viral antigen and antibody kinetics, antibody class, and reagent quality could influence the performance of a serology-based test^6^, so rigorous validation and contextualization is required.

In general, infection by Coronaviruses initiate when the spike (S) protein interacts with its cellular receptor. The spike protein is a type I viral trimeric glycoprotein composed by an N-terminal S_1_ subunit and a C-terminal S_2_ subunit. S_1_ binds to the cellular receptor through the Receptor Binding Domain (RBD). The RBD is a key determinant of host cell tropism and the major target for antibody-mediated neutralization^7^. There is structural conservation and cross-reactivity between SARS-CoV and SARS-CoV-2 RBD’s^8,9^, but less conservation with the RBD of other HuCoV’s, which makes the RBD a good antigen target for developing serologic assays.

Another widely used serologic marker for SARS-CoV-2 previous infection are antibodies against the nucleoprotein (N), a predominant non-structural protein. Antibodies against N are non-neutralizing. A number of studies have described different kinetics for anti-S and anti-N IgG^10–12^, but the impact of such differences in the interpretation of serological surveys remains to be determined.

Most COVID-19 vaccines elicit an immune response against the spike protein, whereas natural infection by SARS-CoV-2 induces a general immune response against several viral proteins, indicating that the use of different serological markers at a population level can provide relevant information of the vaccination efforts. We report an adapted version of the SARS-CoV-2 anti-S IgG ELISA described by Amanat et al.^13^ as a single-step method for anti-RBD IgG antibody detection. We evaluated and compared its diagnostic accuracy with a commercial semi-automated method for anti-nucleoprotein IgG antibody detection in a set of 438 serum samples from pre-pandemic and confirmed COVID-19 cases. Furthermore, to define both markers baseline seroprevalence prior to the initiation of nation-wide vaccination, we used and compared both methods in parallel in 9068 serum samples from a nationwide COVID-19 serological survey implemented during August–November 2020.

## Results

### Serology tests validation

We collected and analyzed 438 serum samples from 391 RT-PCR confirmed COVID-19 cases, of which 43 had paired samples, one taken during acute disease and the other during convalescence, and two cases had two serial convalescent samples. The sociodemographic characteristics of control and COVID-19 donors are shown in Supplementary Figure S1.

As an initial approach to measure diagnostic accuracy of the IgG anti-RBD ELISA, we performed *Receiver Operating Characteristic* (ROC) curve analysis in cases and controls, according to weeks post-symptom onset (PSO) and estimated the area under the ROC curve (AUC) and 95% confidence intervals as indicators of diagnostic performance (Supplementary Table S1 and Supplementary Fig. S2). AUC did not significantly increased from > 1-week PSO (AUC = 0.94, 95% CI 0.92–0.97) to > 7-weeks PSO (AUC = 0.97, 95% CI 0.95–1.0). These results indicate that AUC for the anti-RBD IgG ELISA is critically dependent on weeks PSO, but from > 1-week PSO high specificity and sensitivity can be achieved.

We compared the AUC of anti-RBD IgG ELISA *versus* anti-Nucleoprotein Elecsys®. Although AUC values were slightly higher for Elecsys®, no statistically significant difference was found at any of the time PSO categories (Supplementary Table S2 and Supplementary Fig. S3). Agreement between both methods was high. In all samples, regardless of time PSO, there was a 91.1% agreement with a Cohen’s Kappa = 0.81 (*P* =  < 0.001) (Supplementary Table S3), which corresponds to an almost perfect agreement^14^.

It is evident that the pattern of both methods ROC curves is different and suggested differences, particularly at higher specificities and earlier PSO (Supplementary Fig. S3). The partial AUC (pAUC) for both methods was calculated at the 1.0–0.9 specificity interval^15^ (Supplementary Table S4). As suggested by the differences in ROC curve pattern, pAUC was larger for Elecsys® in earlier times PSO, indicating that in terms of sensitivity, the Elecsys® system performs slightly better than the RBD ELISA at earlier times PSO.

Finally, we determined the absorbance cut-off for the anti-RBD IgG ELISA. By the time the current work was performed, we estimated a national seroprevalence of 25%, indicating that a highly specific method was required to obtain optimal positive predictive value^16^. Thus, we defined the absorbance cut-off as the lowest value to achieve ≥ 0.99 specificity. At 0.4 absorbance, we obtained the best sensitivity without lowering specificity below 0.99. Consistent with the ROC analysis, sensitivity, negative predictive value (NPV) and Negative Diagnostic Likelihood ratio (NDLR) were deeply affected by time PSO (Supplementary Fig. S4A-C) being reliable until > 3-weeks PSO (Table 1).

**Table 1:** Comparative diagnostic performance of Elecsys® and IgG RBD ELISA at ≥ 3 weeks PSO.

Parameter	Elecsys®		IgG RBD ELISA		*P*	Test	Refs.
	Est	95% CI	Est	95% CI			
Sensitivity	0.925	(0.889–0.961)	0.905	(0.865–0.946)	0.31	Mcnemar	^37^
Specificity	0.994	(0.985–1.000)	0.994	(0.985–1.000)	1.0	Mcnemar	^37^
PPV	0.994	(0.984–1.000)	0.994	(0.983–1.000)	0.98	Generalized Score Statistic	^38^
NPV	0.929	(0.895–0.963)	0.912	(0.874–0.950)	0.32	Generalized Score Statistic	^38^
PDLR	184.2	(26.0–1301.8)	180.2	(25.5–1274.1)	0.98	DLR regression model	^39^
NDLR	0.074	(0.045–0.121)	0.094	(0.06–0.145)	0.31	DLR regression model	^39^

As for specificity, both methods had the same specificity (0.995), as only one out of 199 pre-pandemic samples were false-positive (different sample). As a result, Positive Predictive Value (PPV) and Positive Diagnostic Likelihood ratio (PDLR) were also optimal and largely unaffected by time PSO (Table 1 and Supplementary Fig. S4D–F).

### IgG anti-N and anti-RBD comparative results in the National Health and Nutrition Survey

Anti-RBD National seroprevalence was 23.6% (CI 95% 21.5, 25.6), which is slightly lower (1.3%) than the one observed for anti-N IgG ^16^. National seroprevalence for both markers was 19.7% (CI 95% 18.3, 21.3) (Table 2). Anti-N single-positive seroprevalence was 3.72% (CI 95% 3.1, 4.34) and anti-RBD single-positive seroprevalence was 1.98% (CI 95% 1.58, 2.38). Among seropositives to either markers, 77.7% were double positives, 14.1% were single positive to anti-N IgG and 8.1% were single positive to anti-RBD IgG.

**Table 2:** Seroprevalence (%) of anti SARS-CoV-2 IgG in México according to sociodemographic characteristics.

	Anti-N and -RBD					Anti-RBD*				
	n	N	%	95% CI	%*	n	N	%	95% CI	Δ S–N (%)
**National**	9068	125	19.7	18.3, 21.3	23.6	9068	125	23.6	21.5, 25.6	-1.3
**Age (years)**										
1–9	655	19.3	16.6	13.0, 20.8	19.9	655	19.3	19.9	15.3, 24.2	0.8
10–19	1107	21.9	19.7	16.8, 23.0	23.6	1107	21.9	24.3	20.7, 28.2	1.5
20–29	1548	22.7	20.7	18.4, 23.2	24.8	1548	22.7	24.6	21.6, 27.8	-1.4
30–39	1304	15.9	21.5	18.8, 24.4	25.8	1304	15.9	25.5	22.1, 29.1	-0.5
40–49	1490	17.7	24.7	21.8, 27.8	29.6	1490	17.74	29.3	25.8, 32.9	1.8
50–59	1253	11.5	19.3	16.4, 22.6	23.1	1253	11.5	23.1	19.3, 26.9	-0.4
≥ 60	1711	15.8	15.4	13.3, 17.8	18.5	1711	15.8	17.9	15.2, 20.6	0.4
**Sex**										
Male	3775	60.9	19.5	17.7, 21.5	23.4	3775	60.9	23.3	20.9, 25.9	-2.0
Female	5293	64.0	20.0	18.3, 21.8	24.0	5293	64.0	23.9	21.5, 26.1	-0.6
**Education**										
Elementary school or less	3218	45.8	18.4	16.3, 20.8	22.1	3218	45.8	22.1	19.4, 24.9	-0.1
Middle school	2412	30.8	22.9	20.4, 25.7	27.5	2412	30.8	27.0	23.8, 30.3	-1.3
High school	1745	24.2	20.5	18.0, 23.4	24.6	1745	24.2	24.9	21.4, 28.5	-2.6
Graduate/Posgraduate	1628	21.5	17.5	15.1, 20.1	21.0	1628	21.5	21.0	18.1, 24.3	-1.4
Ocupation status**										
Unemployed	2864	29.6	19.8	17.7, 22.1	23.7	2738	29.1	24.0	21.2, 26.8	0.0
Student	889	15.5	17.6	13.9, 22.2	21.1	591	9.9	21.1	16, 26.2	-1.2
Retired	457	40.3	13.8	10.3, 18.4	16.5	434	4.0	16.2	11.4, 20.9	-0.5
Formal employee***	1526	19.3	22.5	19.9, 25.2	27.0	1468	19.7	26.6	23.5, 29.9	-3.5
Informal employee	2798	33.7	21.8	19.6, 24.1	26.1	2705	33.7	26.1	23.3, 29	-1.9
**Urbanity**										
Rural	2276	28.4	16.9	14.0, 20.2	20.3	2186	26.7	20.1	16.7, 23.6	-1.0
Urban	2895	38.5	21.0	18.1, 24.1	25.2	2808	37.5	25.4	21.7, 29.1	-1.7
Metropolitan	3897	57.9	20.3	18.3, 22.3	24.3	4074	60.8	24.0	21.4, 26.6	-1.2
**Region**										
North-Pacific	899	11.7	24.1	19.6, 29.1	28.9	899	11.7	28.0	22.5, 33.6	-3.0
North-Border	980	16.1	17.2	13.7, 21.4	20.6	980	16.1	21.7	17.1, 26.4	0.7
Central-Pacific	892	13.7	15.6	11.3, 21.2	18.7	892	13.7	19.6	14.1, 25.1	0.2
Central-North	1617	16.1	14.0	11.0, 17.6	16.8	1617	16.1	17.2	13.3, 21.3	-1.9
Center	929	12.3	20.6	17.1, 24.7	24.7	929	12.3	23.8	19.7, 28.2	-1.7
Mexico City	1001	9.1	17.1	13.6, 21.3	20.5	1001	9.1	19.8	15.1, 24.2	0.2
State of Mexico	826	16.8	19.6	15.8, 24.0	23.5	826	16.8	23.5	18.1, 28.6	0.0
South-Pacific	1011	16.0	18.4	14.0, 23.7	22.1	1011	16.0	22.3	17.0, 27.7	-2.0
Peninsula	913	12.9	33.5	28.6, 38.8	40.2	913	12.9	38.2	32.3, 44.3	-4.7
Comorbidity****										
No	6982	103.2	19.6	18.1, 21.3	23.5	6982	103.2	23.5	21.2, 25.7	0.2
Yes	2086	21.7	20.4	17.9, 23.1	24.5	2086	21.7	23.9	20.8, 27.2	0.9

*Adjusted by sensitivity and specificity for Anti-RBD (Table 1) and combined N and RBD.

**15 years or more.

***If you are an employee with access to social services or private medical insurance.

****Includes diabetes, hypertension, obesity, cardiovascular disease, chronic obstructive pulmonary disease, HIV, cancer.

We analyzed the seroprevalence based on positivity to both or single markers according to socio-demographic characteristics (Table 3). The prevalence of double positives was similar across age and sex groups. By region, only Mexico City seemed to have a higher prevalence of double positives (90.4%, 95%CI 85.9, 93.6) compared to the rest of the regions that ranged from 69.7% (95% CI 60.8, 77.4%) in the Central-North to 82.4% (95% CI 77.3, 86.5%) in the Yucatan Peninsula. The prevalence of single-positive anti-N ranged from 9.8% (95% CI 6.9, 13.8%) in the age group 40–49 to 18.5% (95% CI 13.7, 24.5%) in the age group 20–29 years; 17.0% (95% CI 14.2, 30.1%) in males and 12.3% (95% CI 9.8, 15.2%) in females; across regions, Mexico City had the lowest prevalence (4.2%, 95% CI 2.3, 7.4%) and Central-North, the highest (19.6%, 95% CI 14.5, 26.0%). The prevalence of only positive to anti-RBD ranged from 6.2% (95% CI 3.8, 10.0%) in the age group 60 and older to 10.5% (95% CI 6.5, 16.6%) in 10–19 years; males and females had the same prevalence (7.8%); and across regions, Central-Pacific had the highest prevalence (12.3%, 95% CI 6.9, 20.9%) and Peninsula, the lowest (3.4%, 95% CI 2.1, 5.5%).

**Table 3:** Distribution of double-positive (anti-N and anti-RBD), only positive to anti-N, and only positive to anti-RBD by age, sex, region, and symptoms. Ensanut-Covid19, 2020, Mexico.

	Anti-N and anti-RBD		Only anti-N		Only anti-RBD	
	n = 1749		n = 318		n = 183	
	weighted sample = 24.7 mil		weighted sample = 4.6 mil		weighted sample = 2.5 mil	
	%	CI	%	CI	%	CI
**Age (years)**						
0–9	76.9	65.5,85.3	15.0	8.0, 26.6	8.1	4.1, 15.2
10–19	77.4	69.6,83.6	12.1	7.5, 18.8	10.5	6.5, 16.6
20–29	74.4	68.7,79.4	18.5	13.7, 24.5	7.1	4.6, 10.8
30–39	75.8	69.3,81.3	17.4	12.9, 23.0	6.7	3.9, 11.4
40–49	82.8	78.1,86.7	9.8	6.9,13.8	7.4	4.8, 11.1
50–59	75.4	68.8,81.0	16.9	12.2, 23.0	7.6	4.9, 11.7
60 +	81.4	76.0,85.8	12.4	9.0, 17.0	6.2	3.8, 10.0
**Sex**						
Male	75.3	71.6,78.6	17.0	14.2, 20.1	7.8	5.8, 10.3
Female	79.9	76.6,82.8	12.3	9.8, 15.2	7.8	6.1, 10.0
Region*						
North-Pacific	79.3	73.9,83.9	15.3	11.6, 20.0	5.4	2.7, 10.3
North-Border	74.3	66.8,80.7	13.6	9.1, 19.7	12.1	8.0, 17.9
Central-Pacific	74.4	64.2,82.5	13.3	7.9, 21.5	12.3	6.9, 20.9
Central-North	69.7	60.8,77.4	19.6	14.5, 26.0	10.6	6.4, 17.1
Center	81.3	73.1,87.4	13.4	8.9, 19.7	5.3	2.8, 9.7
Mexico City	90.4	85.9,93.6	4.2	2.3, 7.4	5.4	3.1, 9.2
State of Mexico	78.8	69.5,85.8	12.9	6.8, 23.3	8.3	4.6, 14.5
South-Pacific	71.9	63.6,78.9	19.5	13.9, 26.5	8.7	4.6, 15.6
Peninsula	82.4	77.3,86.5	14.2	10.1, 19.6	3.4	2.1, 5.5

*North-Border: Chihuahua, Coahuila, Nuevo León, Tamaulipas; Central-Pacific: Colima, Jalisco, Michoacán; Central-North: Aguascalientes, Durango, Guanajuato, Querétaro, San Luis Potosí, Zacatecas; Center: Hidalgo, Tlaxcala, Veracruz; North-Pacific: Baja California, Baja California Sur, Nayarit, Sinaloa, Sonora; South-Pacific: Guerrero, Morelos, Oaxaca, Puebla; and Peninsula: Campeche, Chiapas, Quintana Roo, Tabasco, Yucatán.

To identify the sociodemographic factors associated with single positivity compared to double positivity we performed a multinomial logistic regression (Table 4). Females, compared to males, had 0.66 times the relative probability of being only positive to anti-N over double-positive (*p* value = 0.01). Compared to Mexico City, all regions had a higher relative probability of being only positive to anti-N over double-positive and the North-Border, Central-Pacific, and Central-North had a higher relative probability of being only positive to anti-RBD over double-positive. No significant differences were observed in seropositivity to either single marker according to self-reported time post-symptom onset.

**Table 4:** Multinomial logistic model for only positive to anti-N and only positive to anti-S compared to double-positive (anti-N and anti-S). Ensanut-Covid19, 2020, Mexico.

	Only anti-N vs. Both			Only anti-RBD vs. Both		
	RRR	95% CI	*p* value	RRR	95% CI	*p* value
**Age (years)**						
0–9	Ref			Ref		
10–19	0.83	0.34, 2.02	0.68	1.40	0.57, 3.40	0.46
20–29	1.33	0.58, 3.02	0.50	0.96	0.41, 2.27	0.93
30–39	1.29	0.56, 2.99	0.55	0.91	0.35, 2.35	0.84
40–49	0.64	0.27, 1.52	0.31	0.86	0.38, 1.94	0.72
50–59	1.28	0.55, 2.99	0.57	1.01	0.42, 2.45	0.98
60 +	0.8	0.36, 1.79	0.59	0.82	0.33, 2.00	0.66
**Sex**						
Male	Ref			Ref		
Female	0.66	0.48, 0.9	0.01	0.98	0.65, 1.49	0.94
Region*						
Mexico City	Ref			Ref		
North-Pacific	4.42	2.19, 8.92	< 0.001	1.12	0.45, 2.81	0.80
North-Border	4.05	1.9, 8.640	< 0.001	2.73	1.31, 5.68	0.01
Central-Pacific	4.18	1.75, 9.95	0.001	2.69	1.13, 6.40	0.03
Central-North	6.39	3.11, 13.12	< 0.001	2.61	1.16, 5.88	0.02
Center	3.81	1.73, 8.39	0.001	1.08	0.43, 2.70	0.87
State of Mexico	3.92	1.56, 9.89	0.004	1.77	0.77, 4.08	0.18
South-Pacific	6.30	2.95, 13.47	< 0.001	2.01	0.84, 4.82	0.12
Peninsula	3.96	1.90, 8.27	< 0.001	0.69	0.32, 1.47	0.33

*North-Border: Chihuahua, Coahuila, Nuevo León, Tamaulipas; Central-Pacific: Colima, Jalisco, Michoacán; Central-North: Aguascalientes, Durango, Guanajuato, Querétaro, San Luis Potosí, Zacatecas; Center: Hidalgo, Tlaxcala, Veracruz; North-Pacific: Baja California, Baja California Sur, Nayarit, Sinaloa, Sonora; South-Pacific: Guerrero, Morelos, Oaxaca, Puebla; and Peninsula: Campeche, Chiapas, Quintana Roo, Tabasco, Yucatán.

## Discussion

Serosurveillance is a highly valuable approach to study epidemic dynamics and to inform public health interventions. As such, the election of the serologic method employed determines the quality of data retrieved. Aiming to validate a useful method for nation-wide serosurveillance, we have analyzed the diagnostic accuracy of an in-house, single-step adaptation of the SARS-CoV-2 RBD IgG ELISA shared by the Krammer laboratory^13,17^.

Once validated with a panel of pre-pandemic controls and RT-PCR-confirmed COVID-19 cases, we further compared the performance of this method with a commercial system for determining anti-N IgG in a nation-wide serosurvey. Our results indicate no significant differences in the performance of either method for serosurveillance, although slight differences in sensitivity were found, mainly related to lower sensitivity of our in-house method at early times post-symptom onset. A plausible explanation for such difference is that Elecsys® is a much more analytically sensitive electro-chemiluminescence immunoassay (ECLIA) that may detect lower quantities of antigen-bound IgG than a standard ELISA. Thus, anti-RBD ELISA test could be missing individuals recently infected, or infected several months ago, when anti-RBD response has waned and the amount of antibody is below limits of detection. In agreement with our results, ECLIA is more sensitive and has a wider linear range for detecting seroconversion against malarial antigens^18^.

The in-house SARS-CoV-2 RBD IgG ELISA method has several advantages for a middle-income country, such as Mexico. First, it can be implemented using standard equipment, which is usually available at serology labs. Second, reagents are low cost and can be more easily produced and /or purchased than proprietary kits, reducing the economic burden of surveillance. For the whole serosurvey, we used ~ 1 mg of recombinant RBD. Finally, adaptations can be made to increase the efficiency and reduce waste. In our case, we further simplified the original method by obviating the second confirmatory ELISA that uses the recombinant stabilized S protein, which represented a reduction in cost and time.With these changes, a validation study was performed for both tests to evaluate their diagnostic performance and to assess it in the specific context of Mexico, prior the introduction of COVID-19 vaccination. Endemic CoV’s have been present in Mexico and are frequently detected during flu season; also, Mexico is endemic for vector-borne diseases such as dengue, Zika and malaria. CoV’s and malaria have shown some degree of cross-reactivity with SARS-CoV-2 and could affect the specificity of the in-house method^19–21^; thus, a context-specific validation was needed. Given our concern with specificity, we assembled a large panel of controls from all regions of the country, using historical blood samples, allowing for a wide representation of prior viral exposures. The diagnostic accuracy of our adapted RBD IgG ELISA is comparable to what has been previously described by Indenbaum et al., in Israel who performed the adaptation of the same method shared by the Krammer lab, and showed no additional gain in sensitivity by the inclusion of IgA or IgM class detection^22^.

As reported by manufacturer, Elecsys® system has an overall specificity of 0.998 (95%CI 0.996–0.999)^23^. In an in-house validation with samples of Mexican origin, both methods displayed excellent specificity (0.995, 95% CI 0.985–1.0), ensuring high PPV in low prevalence settings (i.e. early pandemic phase, rural areas, etc.). The high specificity observed suggests that cross-reactivity in Mexico is low, increasing our confidence in the assessment, however SARS-CoV-2 serosurveillance in malaria-endemic foci along the Mexican Pacific coast, Central and South America should be interpreted carefully.

Apart from the diagnostic accuracy of each method, there is evidence that both tests provide qualitatively different information. Anti-RBD IgG titers correlate better with SARS-CoV-2 neutralization than anti-N titers^10,11,24^, and antibody decay seems slower for anti-S than for anti-N IgG^12,25,26^. Interestingly, children are often asymptomatic and in general less affected than adults^27^, and make more robust anti-S IgG antibody response than for the anti-N IgG SARS-CoV-2^28^, suggesting that the sensitivity of N-based IgG immunoassays could be lower in children. Moreover, asymptomatic and mild COVID-19 adults make less robust antibody responses against SARS-CoV-2^10,29–31^. A limitation of the validation approach used in this work is that all COVID-19 samples included in the panel were only from symptomatic adults, potentially underestimating false negatives derived from asymptomatic adult and children infections in large-scale serosurveillance.

Despite differences of both methods in the antibody kinetics, age differences, and analytical sensitivity differences, the results obtained at a nation-wide survey are remarkably similar for seroprevalence estimation. Nevertheless, the impact of single-marker seropositivity at an epidemiological scale is not negligible. We can think of two broad explanations of single positivity as a result of natural infection in non-vaccinated individuals:

(1) True single positivity, implying that for some biological reason, some individuals seroconvert to only one marker. As discussed, predominant seroconversion to S over N has been described in children, presumably because less viral replication favors immune response against structural antigens (i.e. Spike) over non-structural proteins such as (N)^28^. Although tested, we did not find any association between single S positivity and age. A matter of sample size (14 cases with single S positivity of a total of 1405 children under 9 years old) could explain lack of association.

(2) False single positivity: Individuals that seroconverted to both markers, but at the time of sampling, only one marker came out positive. In this case, different kinetics, test sensitivity or both could explain such scenario. Different kinetics of anti-N and anti-S assessed during the first pandemic waves in longitudinal cohorts for up to 8 months is well documented, indicating as a more rapid waning of anti-N antibodies than anti-S^12^, which could explain at least some cases of single anti-S positivity. However, our regression model failed to reveal any association with single positivity and self-reported number of months after infection.

As for test sensitivity, in our test validation we described that anti-S is slightly less sensitive than anti-N in early times PSO (not statistically significant). However, the calculation of partial AUC at high specificity values yielded significant differences in partial AUC’s (Supplementary Table S2). Thus is possible that the in-house anti-S test may be missing true positives in early phase of infection, contributing to anti-N single positivity. Not surprisingly, our regression model also failed to reveal any association between anti-N single positivity and months after infection, because seroconversion is measured in days/weeks, not months. Women have a distinctive qualitative and quantitative antibody response to influenza vaccination^32^. The observed differences in single-positivity to anti-RBD IgG in women are intriguing and could be the result immune response sex dimorphism. In addition, it should be noted that differences obtained in single-positivity to serological markers could also be the result from differences in the time of survey sampling and time since infection, which was different according to the phase of the epidemic wave in which each region was at the time of the survey.

Beyond the validation of the diagnostic accuracy of anti-N and anti-S IgG, this work also provides a useful baseline of seroreactivity based on natural infection. As COVID-19 vaccination coverage increases and novel SARS-CoV-2 variants are selected, seroprevalence estimation will have to take into account that most vaccines elicit anti-spike antibodies only. Thus, the combination of anti-N and anti-S serological markers may be the only approach to discriminate between natural infection and vaccination-induced seroconversion.

In conclusion, the in-house single-step anti-RBD IgG ELISA is a simple, economic, and robust assay with optimal sensitivity and specificity, with a comparable diagnostic performance in a population seroprevalence survey than the anti-N Elecsys® commercial system. As global vaccination coverage increases, combining both serological markers could be more informative in public health decisions than a single test alone.

## Methods

### COVID-19 cases and control serum panel for anti-RBD and N IgG test validation

Between April and August 2020, we prospectively recruited participants 18 years of age and older, with clinical presentation compatible with COVID-19 and hospitalized in preselected Mexican Institute for Social Security (IMSS) clinics of the Mexico City metropolitan area. Participants provided written informed consent. All methods were approved and performed according to the Research Ethics board of the Instituto Mexicano del Seguro Social (IMSS) guidelines (R-2020-785-065). For those who agreed to participate in the study, a serum sample was taken as soon as the RT-PCR confirmation was available. A second serum sample was taken > 3 weeks later, when possible. For validation, only RT-PCR + samples were analyzed, irrespectively of viral load. Asymptomatic individuals, or those that did not recalled their date of symptom onset were excluded. After blood draw, samples were centrifuged, and the serum was stored at − 80 °C. As non-COVID-19 samples (controls), we used a collection of 199 serum samples stored at − 80 °C, obtained during the National Health survey (ENSANUT 2018) in 2018 when no circulation of SARS-CoV-2 existed. According to the geographical origin of samples, control samples are countrywide representative, whereas COVID-19 samples derive from the Mexico City metropolitan area (MCMA) (Supplementary Fig. S1A). Age and gender distribution between cases and controls was comparable (p-value = 0.08) (Supplementary Fig. S1B–C), despite a deliberate enrichment of children samples in pre-pandemic controls to address potential antibody cross-reactivity with seasonal human coronavirus infection, which are common in the younger population^33^.

### Anti-RBD IgG ELISA

The first step (anti-RBD screening) of the SARS-CoV-2 protocol was used as reference for a single-step in-house protocol^13^, and only minor modifications due to reagent and material availability were implemented. The plasmid encoding SARS-CoV-2 RBD was kindly provided by Dr. Florian Krammer at Mount Sinai Medical Center and stably transfected in CHO cells and cryopreserved. For large scale production (50 L), CHO cells were grown serially for four days at 37 °C, 5% CO_2_ and 130 rpm agitation in 1000-mL shake flasks (200 mL culture volume), then seeded in 5 L New Brunswick bioreactor for 3 additional days at similar conditions and with dissolved oxygen tension controlled > 30% of saturation. Finally, 3.7 L were further transferred to a 200 L Biostat CultiBag bioreactor and cultured for one more week. Fifty L of supernatant were depth-clarified and concentrated to 4 L by filtration with a Cogent M1 ultrafiltration system and a membrane cutoff of 10 kDa. Concentrated supernatant was subjected to affinity purification with Ni column in an AKTA prime chromatograph and further dialysis and sterile filtration with Millipak 20 filters. Final RBD purity was above 80% as quantified by SDS-PAGE.

RBD was immunoadsorbed to High bind 96-well ELISA flat bottomed plates (Corning CLS9018) at 2 μg/mL in PBS at 4 °C overnight and washed manually in PBS-Tween 20, followed by blocking at 4 °C in PBS-5% milk (Difco) for two hours at 4 °C. After washing, plates with 1:50 diluted serum samples were incubated at RT for two hours. After washing, plates were incubated with a 1:5,000 dilution of HRP-conjugated goat anti-human IgG (Sigma) for 1 h at RT, washed and developed with OPD in Phosphate/Citrate buffer. Absorbance was read in an ELISA plate reader (Biotek ELx808) with a 450 nm filter. Background absorbance was the average absorbance of 16 blank wells, which was subtracted to each sample well.

### Anti-N IgG electro-chemiluminescence immunoassay (ECLIA)

Detection of anti-Nucleoprotein IgG was performed with the Elecsys® Anti-SARS-CoV-2 Electro-chemiluminescence immunoassay (ECLIA) (Roche Diagnostics), using the Cobas e 411 analyzer, according to manufacturer’s instructions.

### Statistical analysis of diagnostic performance

Antigen-specific class-switched antibody secretion and seroconversion are highly dependent of the time since antigen exposure up to the development of functional germinal centers^25^. To account for the time of exposure, we categorized COVID-19 cases according to weeks PSO (Supplementary Fig. S1 and Supplementary Table S2) and evaluated diagnostic accuracy by estimating different parameters of diagnostic performance such as AUC of the ROC curve, and partial AUC using the R package pROC^34^. For statistical comparison of AUC and partial AUC we used the DeLong^35^ and Bootstrap methods^36^, respectively. ROC analysis allowed us to define the optical density (O.D.) cutoff for anti-RBD ELISA based on an expected specificity of ≥ 0.99.

To estimate sensitivity, specificity, predictive values and diagnostic likelihood ratios at different weeks PSO and to compare the performance of anti-RBD ELISA with the Elecsys® Anti-Nucleoprotein ECLIA, we used the statistical R package DTComPair^37^. Differences in sensitivity and specificity between methods were analyzed using the McNemar test^38^. Differences in predictive values were evaluated with the Generalized Score Statistic^39^. Diagnostic likelihood ratios were evaluated with the DLR regression model^40^. The concordance between both methods was estimated using Cohen’s Kappa statistic^41^ and Maxwell’s RE coefficient^42^ with the R package irr^43^.

### Nationwide serological survey

The National Health and Nutrition survey (ENSANUT 2020-COVID-19) is a nationwide probabilistic household survey based on multistage and stratified sampling of participating households, implemented during August to November 2020. It included 10,216 complete household interviews and 9464 serum samples for biochemical markers and COVID-19 serology^44^. Participants provided written informed consent as approved by the Research ethics board of the National Institute of Public Health (CI 1679), and all methods were performed according to the Declaration of Helsinki and corresponding Institutional guidelines. All serum samples were independent of the validation sample set and were processed with the Elecsys® Anti-SARS-CoV-2 Electro-(ECLIA), whereas 9,068 were processed with the anti-RBD IgG ELISA.

### Statistical analysis of anti-N and anti-RBD seroprevalence

We calculated the prevalence with 95% confidence intervals of only anti-RBD, only anti-N and both anti-RBD and anti-N, overall and by sociodemographic characteristics. Prevalence was adjusted by sensitivity and specificity of each validation test^45^. We grouped seropositive individuals as double-positive (anti-N and anti-RBD), positive only to anti-N, and positive only to anti-RBD. We calculated the distribution by age, sex, region, and symptoms with 95% confidence intervals. We then used a multinomial logistic regression including age, sex, and region, using the three groups as the outcome. We report the exponentiated coefficients as relative probabilities of only anti-N and only anti-RBD, using double positive as the reference group. We used the survey commands to account for the survey design. The analysis was made in Stata v14 (StataCorp).

## Supplementary Information

Supplementary Information.
