# Supplementary material for: Comparable diagnostic accuracy of SARS-CoV-2 Spike RBD and N-specific IgG tests to determine pre-vaccination nation-wide baseline seroprevalence in Mexico
Source: Sci Rep. 2022 Oct 26;12:18014. doi: 10.1038/s41598-022-22146-8 (PMC9606250; doi:10.1038/s41598-022-22146-8)
Supplement: Supplementary file 1 — Supplementary Information. [file 41598_2022_22146_MOESM1_ESM.pdf]

# Supplementary information

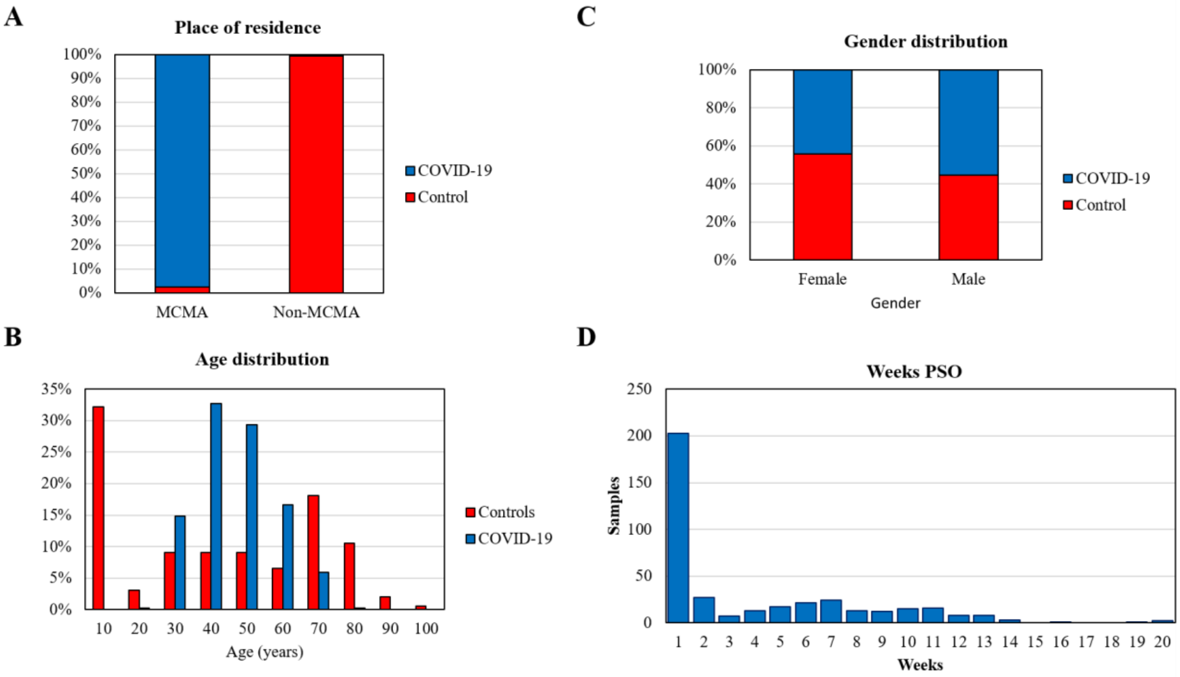


**Figure S1. Sociodemographic characteristics and clinical evolution of serum donors for the validation panel**. According to place of residence (A), age distribution (B), gender (C) and weeks post-symptom onset (PSO) of COVID-19 confirmed cases (D). Pre-pandemic serum samples (negative controls) are in red bars and COVID-19 cases are in blue bars. Mexico City Metropolitan Area (MCMA).

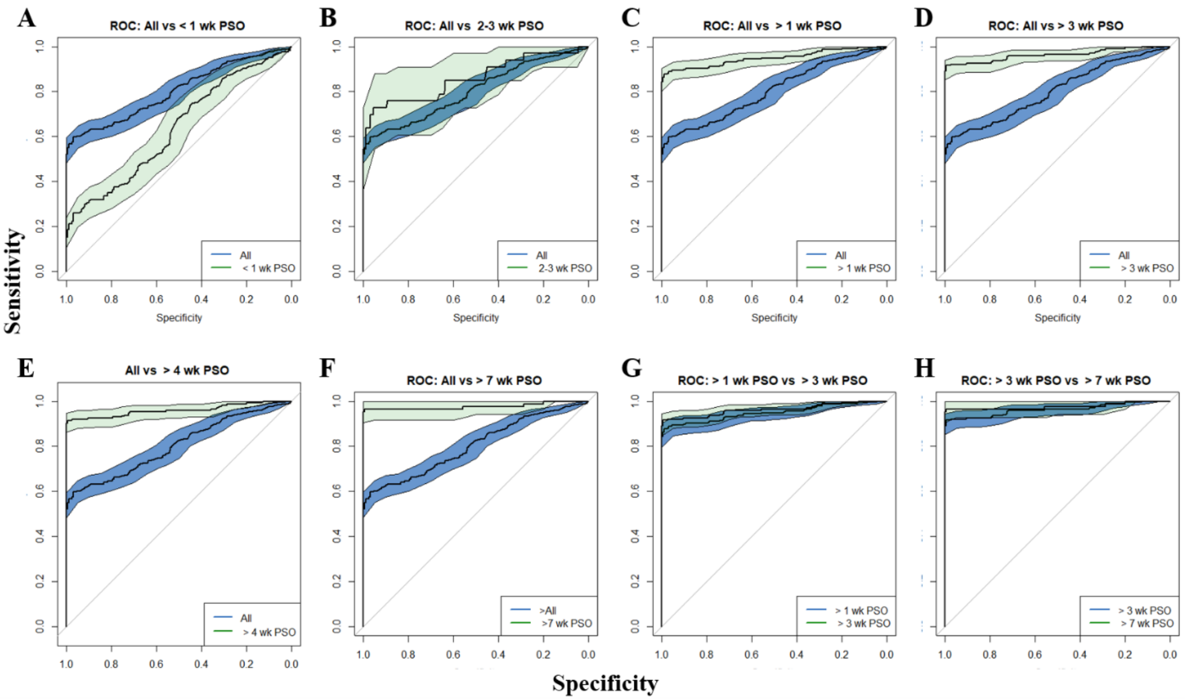


**Figure S2. Comparison of Area Under the *Receiver Operating Characteristic* (ROC) curve of IgG anti-RBD ELISA at various days post-symptom onset (PSO).** *ROC* comparison using all COVID-19 confirmed cases (regardless PSO) as reference at various times (weeks) post-symptom onset. Sensitivity confidence intervals (De Long) are shown in light green for each PSO and in blue for all (A-F). Analysis and graphs were generated with pROC [33]. Detailed ROC parameters and statistical comparisons are shown in Supplementary Table S1.

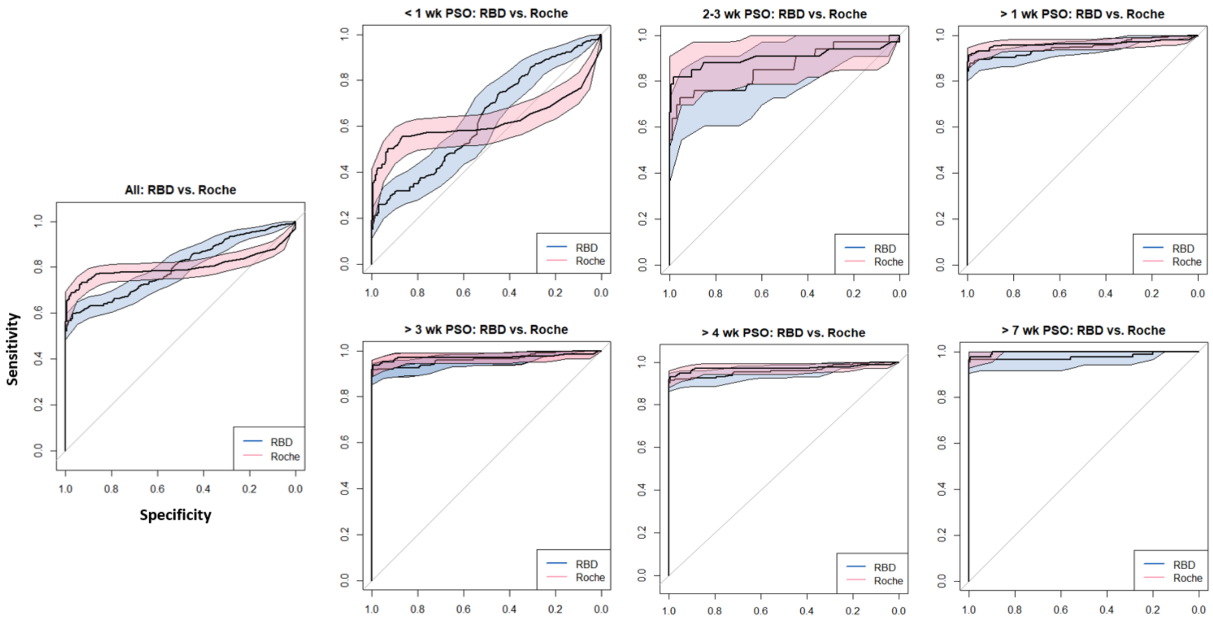


**Figure S3. Area Under the *Receiver Operating characteristic* curve of RBD *vs.* Elecsys®.** Comparison of ROC curves of anti-RBD ELISA vs. Elecsys® at various time points PSO. Blue and pink Sensitivity CI (De Long) correspond to anti-RBD ELISA and Elecsys®, respectively. Analysis and graphs were generated with pROC [34]. Detailed ROC parameters and statistical comparisons are in Supplementary Table S2.

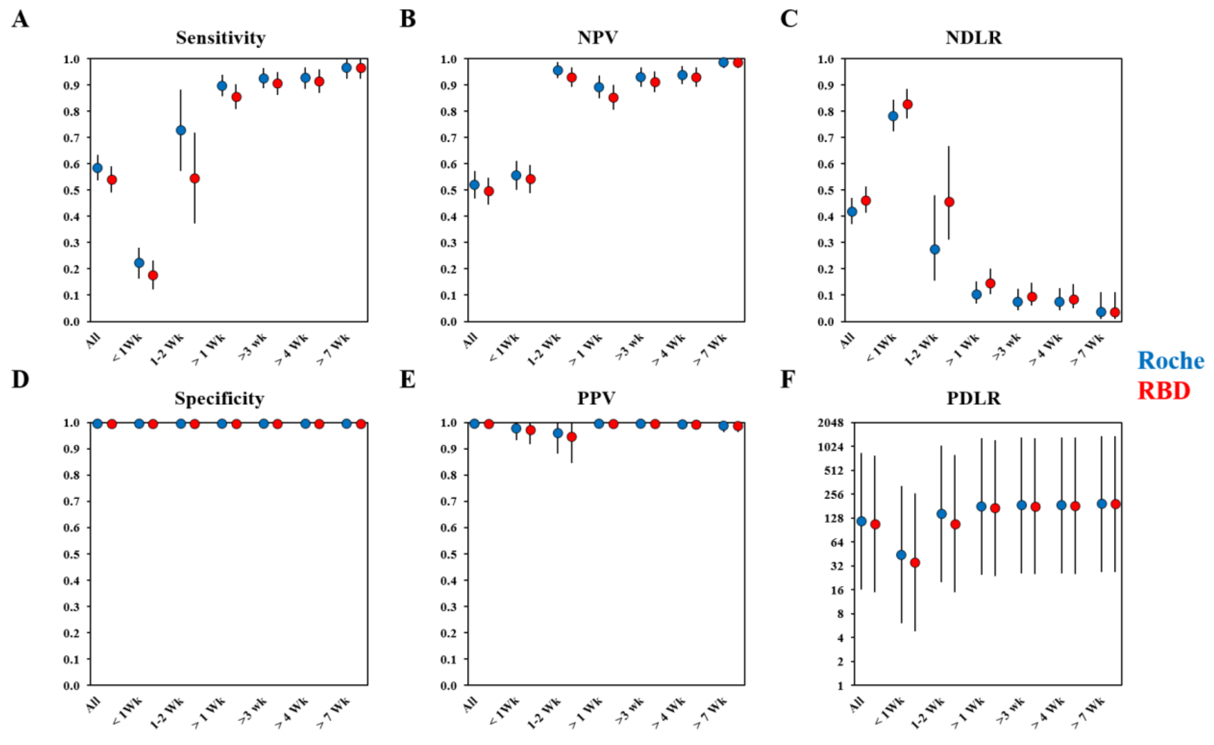


**Figure S4. Diagnostic accuracy of anti-RBD ELISA and Elecsys® at different time points PSO**. Sensitivity (A), Negative predictive value (B), Negative diagnostic likelihood ratio (C), Specificity (D), Positive predictive value (E) and Positive diagnostic likelihood ratio (F). Elecsys® (Roche. Blue dots) and anti-RBD ELISA (red dots).

**Supplementary Table S1. Comparison of Area Under the *Receiver Operating Characteristic* Curve of IgG anti-RBD ELISA at various days post-symptom onset (PSO)**

| Reference | AUC Ref | CI Ref | Comparator | AUC comp | CI Comparator | pval |
|---|---|---|---|---|---|---|
| All | 0.804 | 0.771-0.836 | < 1 wk PSO | 0.636 | 0.582, 0.69 | < 0.001 |
| | | | ≥ 1 wk PSO | 0.949 | 0.927, 0.971 | < 0.001 |
| | | | 1-2 wk PSO | 0.858 | 0.769, 0.947 | 0.26 |
| | | | ≥ 3 wk PSO | 0.963 | 0.943, 0.983 | < 0.001 |
| | | | ≥ 4 wk PSO | 0.961 | 0.938, 0.983 | < 0.001 |
| | | | ≥ 7 wk PSO | 0.977 | 0.95, 1.0 | < 0.001 |
| ≥ 1 wk PSO | 0.949 | 0.927-0.971 | ≥ 3 wk PSO | 0.963 | 0.943, 0.983 | 0.33 |
| ≥ 3 wk PSO | 0.963 | 0.943-0.983 | ≥ 7 wk PSO | 0.977 | 0.95, 1.0 | 0.42 |

**Supplementary Table S2. Area Under the *Receiver Operating characteristic* curve of RBD vs. Elecsys®**

| Weeks PSO | Cases | Controls | AUC RBD | CI RBD | AUC Elecsys® | CI Elecsys® | P val | Power |
|---|---|---|---|---|---|---|---|---|
| All | 438 | | 0.80 | 0.77-0.83 | 0.80 | 0.77, 0.83 | 0.99 | 0.03 |
| < 1 wk | 203 | | 0.64 | 0.58-0.69 | 0.62 | 0.56, 0.68 | 0.66 | 0.09 |
| 1-2 wk | 33 | | 0.86 | 0.76-0.94 | 0.90 | 0.81, 0.99 | 0.33 | 0.16 |
| ≥ 1wk | 235 | 199 | 0.95 | 0.92-0.97 | 0.96 | 0.94, 0.98 | 0.26 | 0.20 |
| ≥ 3 wk | 202 | | 0.96 | 0.94-0.98 | 0.97 | 0.95, 0.99 | 0.46 | 0.12 |
| ≥ 4 wk | 176 | | 0.96 | 0.93-0.98 | 0.97 | 0.95, 0.99 | 0.32 | 0.14 |
| ≥ 7 wk | 85 | | 0.98 | 0.95-1.0 | 1.00 | 0.99, 1.0 | 0.12 | 0.11 |

**Supplementary Table S3. Agreement between RBD ELISA and Elecsys®**

| | N | %-agree | Cohen's Kappa | p | Maxwell's RE |
|---|---|---|---|---|---|
| All | 637 | 91.1 | 0.81 | 0.0 | 0.82 |
| < 1 wk | 402 | 91.5 | 0.52 | 0.0 | 0.83 |
| ≥ 1 wk | 434 | 94.2 | 0.88 | 0.0 | 0.88 |
| 1-2 wk | 232 | 95.7 | 0.75 | 0.0 | 0.91 |
| ≥ 3 wk | 401 | 95.8 | 0.91 | 0.0 | 0.92 |
| ≥ 4 wk | 375 | 96.3 | 0.92 | 0.0 | 0.93 |
| ≥ 7 wk | 284 | 97.9 | 0.95 | 0.0 | 0.96 |

**Supplementary Table S4. Partial Area Under the *Receiver Operating Characteristic* curve (1-0.9 specificity) of RBD vs Elecsys®**

| **Weeks PSO** | **C19** | **Control** | **pAUC RBD** | **pAUC Elecsys®** | **p value** |
|---|---|---|---|---|---|
| All | 438 | 199 | 0.059 | 0.070 | **7.07E-07** |
| < 1 wk | 203 | 199 | 0.025 | 0.043 | **8.43E-07** |
| 1-2 wk | 33 | 199 | 0.069 | 0.081 | 0.10 |
| ≥ 1wk | 235 | 199 | 0.089 | 0.093 | 0.03 |
| ≥ 3 wk | 202 | 199 | 0.092 | 0.094 | 0.13 |
| ≥ 4 wk | 176 | 199 | 0.092 | 0.094 | 0.20 |
| ≥ 7 wk | 85 | 199 | 0.096 | 0.098 | 0.54 |
